# Self-Rated Health Status and Cardiorespiratory Fitness in a Sample of Schoolchildren from Bogotá, Colombia. The FUPRECOL Study

**DOI:** 10.3390/ijerph14090952

**Published:** 2017-08-23

**Authors:** Robinson Ramírez-Vélez, Carolina Silva-Moreno, Jorge Enrique Correa-Bautista, Katherine González-Ruíz, Daniel Humberto Prieto-Benavides, Emilio Villa-González, Antonio García-Hermoso

**Affiliations:** 1 Centro de Estudios para la Medición de la Actividad Física (CEMA), Escuela de Medicina y Ciencias de la Salud, Universidad del Rosario, Bogotá DC 111221, Colombia; carolinasilva1111@gmail.com (C.S.-M.); jorge.correa@urosario.edu.co (J.E.C.-B.); 2 Grupo de Ejercicio Físico y Deportes, Vicerrectoría de Investigaciones, Universidad Manuela Beltrán, Bogotá DC 110231, Colombia; katherine.gonzalez@docentes.umb.edu.co (K.G.-R.); danielprietob@gmail.com (D.H.P.-B.); 3 Department of Education Sciences, University of Almería, 04120 Almería, Spain; evilla@unach.edu.ec; 4 PROFITH “PROmoting FITness and Health through Physical Activity” Research Group, Department of Physical Education and Sport, School of Sport Sciences, University of Granada, 18010 Granada, Spain; 5 Laboratorio de Ciencias de la Actividad Física, el Deporte y la Salud, Universidad de Santiago de Chile, Facultad de Ciencias Médicas, USACH, Santiago 9160030, Chile; antonio.garcia.h@usach.cl

**Keywords:** physical activity, sedentary life style, subjective perception of health, pain, fitness

## Abstract

To evaluate the relationship between Self-Rated Health (SRH) and cardiorespiratory fitness (CRF) in a sample of children and adolescents enrolled in official schools in Bogotá, Colombia. A cross-sectional study was performed with 7402 children and adolescents between 9 and 17 years of age. Participants were asked to rate their health based on eight validated questions, addressing the participants propensity for headache, stomach-ache, backache, feeling-low, irritability/bad mood, nervousness, sleeping-difficulties, and dizziness. The choices were “rarely or never”, “almost every month”, “almost every week”, and “more than once a week/about every day”. Participants performed the international *course-navette* shuttle run test to estimate CRF, and cut-off points for age and gender were used to categorize the healthy/unhealthy fitness zone according to the FITNESSGRAM^®^ criteria. Overall, 16.4% of those surveyed reported a perception of irritability/bad mood “more than once a week/about every day”, followed by feeling-low and nervousness (both with 9.9%). Dizziness had the lowest prevalence with a percentage of 6.9%. Unhealthy CRF in boys increased the likelihood of headaches by 1.20 times, stomach aches by 1.31 times, feeling-low by 1.29 times, nervousness by 1.24 times, and dizziness by 1.29 times. In girls, unhealthy CRF increased the likelihood of headaches by 1.19 times, backache by 1.26 times, feeling-low by 1.28 times, irritability/bad mood by 1.17 times, sleeping-difficulties by 1.20 times, and dizziness by 1.27 times. SRH was associated with CRF in both genders. Early identification of children and adolescents with low CRF levels will permit interventions to promote healthy behaviors and prevent future diseases during adulthood.

## 1. Introduction

Self-Rated Health (SRH) is a relevant construct for a healthy lifestyle and is used to capture a person’s perception of their overall health status [1]. Epidemiological studies and health surveys highlight perceived heath or SRH, defined as the self-assessment that the population or an individual makes of their health condition as a product of their knowledge and own interpretations without necessarily representing a clinical diagnosis by the professional health staff [2,3]. Normally, this indicator is used in population surveys and in censuses of small populations or very specific collective groups [4]. Although, this measurement is subjective, previous studies have shown a close relation between SRH and other physical and mental health outcomes [3,4].

The association between physical fitness and SRH among adolescents may differ according to sex and overall level of physical activity, i.e., the association is stronger among boys and among more physical inactive adolescents [5]. Individuals’ perceptions of their health incorporate psychological, biological, and social dimensions that are unavailable to the external observer, but also provide a dynamic assessment of their current health status while integrating their health history [5]. For example, Idler and Benyamini [6] showed in a narrative review including 27 observational studies that SRH was related to objective health and was conditioned by the recent evolution of individual skills. In populations with poor access to health services, such as school-aged children, SRH studies have been better at showing the burden of the disease than medical records [7]. In the early stages, a study of 1155 Swedish children between 6 and 13 years of age by Petersen et al. [8], found that 23% of the children self-reported weekly recurring headaches, 19% stomach aches, and 18% back pain. In Colombia, Higuita-Gutiérrez et al. [9] performed a study with 3452 adolescents and highlighted that 10% of participants reported eye diseases, 3.1% participants reported diseases of the respiratory system, and 1.1% participants reported diseases of the musculoskeletal system. 

In addition, SRH has been associated with low socioeconomic level, gender (women), lower education level, use of medical services, and poor lifestyle [10,11]. Regarding lifestyle, Kujala et al. [12] stated that healthy lifestyles influence an individual’s perceived health, which is why its absence could be related to the early onset of physical symptoms and future diseases. Thus, a relationship has been described between SRH and the physical condition related to health [13], suggesting that an active lifestyle as well as good cardiorespiratory fitness probably increase self-rated health.

Physical fitness, especially the cardiorespiratory component, is considered an important health indicator in children and adolescents [14]. Previous studies by Janz et al. [15] and Castillo-Garzón et al. [16] showed that a low cardiorespiratory fitness (CRF) level during childhood was associated with a higher risk of cardiovascular and metabolic disease during adulthood. Moreover, it has been described that a lower CRF level in children and adolescents from 9 to 17 years of age is a predictive factor of physical well-being in school-aged children [17,18]; hence, including this health indicator in epidemiological surveillance systems at the educational level is clearly justified [19].

Similarly, a cross-sectional study carried out by Herman et al. [20] with Canadian school-aged children displayed that higher levels of physical activity are directly related to better SRH and a healthy nutritional status. A study by Becerra et al. [21], which included 264 adolescents from Málaga (Spain), reported an inverse relationship between peak oxygen consumption (VO_2_peak) and symptoms such as pain and/or fatigue (r = −0.40; *p* < 0.001) as well as anxiety and insomnia (r = −0.46; *p* < 0.001). Recently, a prospective study with over 16 years of follow up conducted by Kantomaa et al. [22] in a sample of 7063 Finnish adolescents established that a better SRH was associated with a healthier level of CRF in both sexes.

Greater knowledge of SRH in children and adolescents, as well as its relationship with physical fitness parameters, could prevent future associated health risks in this population. Hence, this study sought to evaluate the relationship between SRH and CRF in a sample of children and adolescents enrolled in official schools in Bogotá, Colombia participating in the FUPRECOL (Asociación de la fuerza prensil con manifestaciones de riesgo cardiovascular tempranas en niños y adolescentes colombianos in Spanish) study. We hypothesized that high levels of CRF are associated with rare or absent symptoms among schoolchildren. 

## 2. Methods

### 2.1. Study Design and Sample Population 

Schoolchildren included in this secondary analysis are part of The FUPRECOL Study carried out in Bogotá, Colombia (*n* = 10,000 school children). We selected 27 schools, which already had collaboration agreements established with our research center and therefore were selected primarily for pragmatic, budgetary, and logistical reasons. The FUPRECOL Study methodology has been published elsewhere [23,24]. Data were collected from 2013 to 2016 and the analysis was done in 2016. In this study, we included a sub-sample (*n* = 7404) of 9–17.9-year-old healthy Colombian children and adolescents (girls, *n* = 4124 [55.9%] and boys, *n* = 3280). The children and adolescents belonged to low to middle socioeconomic statuses (SES, 1–3 defined by the Colombian government), enrolled in public elementary and high schools (grades 5 through 11), and from the capital district of Bogota in a municipality in the Cundinamarca Department in the Andean region. The exclusion criteria included having a clinical diagnosis of cardiovascular disease, having Type 1 or Type 2 diabetes mellitus, being pregnant, using alcohol or drugs, and not having lived in Bogota for at least one school year. Exclusion from the study was made effective a posteriori, without the students being aware of their exclusion, to avoid any undesired situations. 

### 2.2. Measurement of SRH

The Health Behavior in School-Aged Children (HBSC) survey is a cross-national research study conducted in collaboration with the World Health Organization Regional Office for Europe, aiming to gain new insight into, and increase the understanding of young people’s health and well-being, health behaviors, and their social context [25]. In this study, we used the SRH-HBSC [25], which addresses the SRH of several symptoms: headache, stomach ache, back ache, low emotional state, irritability or bad mood, nervousness, difficulty sleeping, and dizziness. The responses were restricted to the last six months and the frequency option was distributed into “rarely or never”, “almost every month”, “almost every week”, and “more than once a week/almost every day”. This questionnaire has adequate validity and reliability values in young populations [26]. Nevertheless, in light of the lack of psychometric properties in the Colombian population, a reliability analysis was conducted that revealed internal consistency results (Cronbach’s alpha) of 0.69. Furthermore, in a sub-sample of 229 school-aged children (mean age 12.8 ± 2.4 years, 46.2 ± 12.4 kg, 1.50 ± 0.1 m and 19.9 ± 3.1 kg/m^2^ in the body mass index) and a 7-day period between each test, test-retest (Kappa index) reproducibility values of 0.79 were obtained in the eight questions of the HBSC questionnaire. The surveys were applied to the school-aged children in groups of 20–50 participants in classrooms in order to maintain privacy and freedom in their fulfilment and with the presence of at least two qualified researchers. The questionnaires were completed in approximately 15 min. Prior to applying the questionnaires, the necessary guidelines were provided for their completion, highlighting the need to carefully read the items and the importance of sincerity and anonymity when answering them.

### 2.3. CRF Assessment

Testing procedures were consistent with guidelines for school-based fitness assessments [19]. Tests took place in the school gymnasium or on another available hard surface. CRF was measured using the 20 m shuttle run test as previously described by Léger [27]. Results were recorded to the nearest stage completed. The Léger equation was used to determine VO_2_peak (mL·kg·min^−1^) in each participant [27,28]. The reliability and validity of this test has been widely documented [27,28] and is considered a test of choice for population-based CRF assessments for schoolchildren [19]. All tests were conducted by a trained research team that provided standardized encouragement for participants during all test phases. CRF measurements in a subsample (*n* = 229, median age = 12.8 ± 2.4 years, 46.2 ± 12.4 kg, 1.50 ± 0.1 m, 19.9 ± 3.1 kg/m^2^) were recorded to ensure reproducibility on the day of the study. The reproducibility (R) of our data was R = 0.84. Intra-rater reliability was assessed by determining the intra-class correlation coefficient (ICC = 0.96, 95% CI 0.95 to 0.97) [19].

### 2.4. Anthropometric Variables

Body weight was measured to the nearest 0.10 kg with the participant lightly dressed using a portable electronic weight scale (Tanita^®^ BC544, Tokyo, Japan) with a low technical error of measurement (TEM = 0.510). Body height was measured to the nearest 0.1 cm in bare or stocking feet with the adolescent standing upright against a portable stadiometer (Seca^®^ 274, Hamburg, Germany; TEM = 0.019) [19]. Their body mass index (BMI) was calculated as their body weight in kilograms divided by the square of their height in meters. 

### 2.5. Ethics Statement

The FUPRECOL Study was conducted in accordance with the Helsinki Declaration for Human Studies and approved by the Colombian Data Protection Authority (Resolution 008430/1993 Ministry of Health) and the Review Committee for Research on Human Subjects at the University of Rosario (Code N° CEI-ABN026-000262). All participants were informed of the study’s goals, and written informed consent was obtained from participants and their parents or legal guardians.

### 2.6. Data Analysis

Continuous values were expressed as means and (±) standard deviations and proportions in percentages. Differences were analyzed by Student’s *t*-test or Chi-square test (χ^2^) to explore sex differences. The differences among SRH and the categories of the CRF were tested with the χ^2^ test. To analyze SRH, the five response options were placed into four groups as follows: “rarely or never”, “almost every month”, “almost every week”, and “more than once a week/almost every day”. To analyze VO_2_peak, the CRF variable was recalculated, taking the cut-off point suggested in the 2011 FITNESSGRAM^®^ battery [28]. Therefore, the participants were classified as having a healthy CRF if their VO_2_peak was 40–44 mL·kg·min^−1^ for boys and 38–40 mL·kg·min^−1^ for girls, according to their age. These age-and-sex-specific VO_2_peak cut-off points were validated against the presence of metabolic disorders using representative U.S. data and the 2011 FITNESSGRAM^®^ standards [28]. Finally, a binary logistic regression adjusted by age and BMI was used to determine the association among the CRF categories and the eight questions from the SRH questionnaire. To separate the opinion of participants who saw their self-perceived health as excellent (rarely or never symptoms) from the rest, a group denoted as “sometimes symptoms” was created, combining the responses of “every week” and “more than once a week/about every day”. All analyses were adjusted by age and BMI. Analyses were performed using the Statistical Package for Social Science^®^ software, version 24 (IBM; Chicago, IL, USA), and the level of significance was set to 0.05.

## 3. Results

### 3.1. Descriptive Characteristics

Descriptive statistics for each sex are shown in Table 1. Of the total sample (*n* = 10,000), 7402 school-aged children (74% rate of response) between 9 and 17.9 years of age complied with all the inclusion criteria, of which 4140 (55.9%) belonged to the group of girls. The mean age of the general population was 12.8 (2.3) years, body mass of 44.6 (12.3) kg, height 1.49 (0.12) m, and 19.7 (3.4) kg/m^2^ in the body mass index. The proportion of subjects with low CRF was 31.5%. An unhealthy aerobic capacity was observed in 33.9% of girls and 28.1% of boys (*p* < 0.001).

### 3.2. Self-Rated Health Status

Table 2 presents the distribution of SRH in the sample. Headache and bad mood were the most frequent psychological complaints, with different percentages among boys and girls (*p* < 0.05). Overall, 16.4% reported a perception of irritability/bad mood “more than once a week/about every day”, followed by feeling-low and nervousness (both at 9.9%). Dizziness had the lowest prevalence with a value of 6.9%. In terms of gender, 27.8% of the boys reported a perception of irritability/bad mood “almost every week”, followed by headache at 25.4% and stomach-ache at 20.4%. In girls, the proportion that reported irritability/bad mood “more than once a week/about every day” was at 11.6%, followed by feeling-low at 10.0%.

### 3.3. Relationship among SRH and CRF Categories

Table 3 shows the relationship between SRH and CRF divided in two categories (healthy and unhealthy). There were several significant differences (*p* < 0.05) when comparing the frequency of response between CRF categories among the four categories (“rarely or never”, “almost every month”, “almost every week”, “more than once a week/almost every day), for almost all of the eight SRH dimensions, for both girls and boys. Upon differentiating by gender, girls categorized with unhealthy CRF had a higher frequency of problems “almost every week” and “almost every month” than boys for the eight questions from the SRH questionnaire.

### 3.4. Factors Associated to Low CRF (Unhealthy)

Finally, Table 4 shows the binary regression model to determine the association among the low CRF and the eight questions from the SRH questionnaire. The results show that unhealthy CRF in boys, according to the 2011 FITNESGRAM^®^ criteria, increased the likelihood of reporting headaches by 1.30 times (95% CI 1.09–1.55), stomach aches by 1.31 times (95% CI 1.09–1.58), feeling low by 1.29 times (95% CI 1.08–1.54), nervousness by 1.24 times (95% CI 1.04–1.48), and dizziness by 1.29 times (95% CI 1.05–1.59). In girls, this condition increased the likelihood of headaches by 1.19 times (95% CI 1.04–1.35), backache by 1.26 times (95% CI 1.07–1.48), feeling-low by 1.28 times (95% CI 1.12–1.46), irritability or bad mood by 1.17 times (95% CI 1.03–1.32), sleeping-difficulties by 1.20 times (95% CI 1.03–1.39), and dizziness by 1.27 times (95% CI 1.09–1.48), as shown in Table 4.

## 4. Discussion 

The most relevant finding in this study was that boys and girls categorized with unhealthy CRF had higher odds of problems “more than once a week” and “almost every day” than healthy counterparts in the eight questions from the SRH questionnaire. As should be expected when considering such SRH, the occurrence of subjective physical complaints was relatively low in our study, with irritability/bad mood being more frequently reported, followed by headaches. 

The perception of stomach-ache was higher in girls compared to boys, as previously reported [7]. Subjective indicators of health markedly differ between youth in different countries, although multiple complaints are generally declared at higher proportions in girls compared to boys. From current evidence, the relationship between physical fitness and SRH in a young population is still scarce and confusing, as well as contradictory when comparing both genders [29]. This study found significant differences in the self-reporting of sensed morbidity, finding that girls presented higher prevalence of irritability/bad mood, while boys presented higher prevalence of feeling-low. Prior studies concluded that girls tend to endure 2–3 times more self-reported health problems than boys [29]. Regarding this finding, Johnson and Richter [30] considered that SRH is an adequate indicator of real health, highlighting that girls are often more pessimistic than boys, since they tend to self-report more serious problems related to their health. This fact also coincides with the indirect estimation of CRF because girls with unhealthy CRF showed higher frequencies of somatic problems compared to boys.

A previous study has found relationships between SRH and backache, with low levels of physical activity (<6 h per week) especially in adolescent girls [31], but the physical fitness was not analyzed. In this study, the frequency of reporting experiencing this symptom “almost every day” was higher in girls (4.4% boys, 6.0% girls, *p* < 0.05). The importance of the study of back pain in young people is remarkable since the early appearance of this symptom is a risk factor related to pain in adulthood [32]. Another symptom in the young population that has gained importance in the scientific literature is headache [33], which is additionally associated with poor physical condition [34,35]. In our study, this factor occurs in girls and boys at frequencies of 12.1% and 6.9%, respectively. Reports show that youth with low levels of physical activity have a higher risk of suffering from frequent headaches [34]. In school-aged children, a recent study published by Vierola et al. [35] revealed that infants with low CRF were 1.95 times more prone to suffering from headaches than infants with healthy values of aerobic fitness, independent of physical activity levels. Unfortunately, we were not able to measure variables related to physical activity levels in the FUPRECOL sample. Additionally, increasing sedentary behaviors, like screen time, have been directly associated with greater presence of musculoskeletal pain and lower self-perception of health, especially in males younger than 12 years of age [36]. This finding is relevant and coincides with our results since reports indicate that these types of diseases are associated with symptoms like cephalea, which may even affect the student’s academic performance [37]. 

Other symptoms reported in our study were irritability/bad mood, nervousness, and feeling-low, which to our knowledge have not been studied well in this population nor have their associations with physical fitness. However, we may speculate that the presence of some of these risk factors can be associated with other unmeasured factors related to morbidity, which seem particular to this age stage, characterized by high family and school demands; nonetheless, this requires further research [38]. In this sense, Hernan-Gómez et al. [39] propose that adolescence may bring diminished positive emotions and decreased vital satisfaction with a gradual increase of negative emotions that could affect the SRH. This finding coincides with that reported by Urzúa [40], who wrote that adolescent girls have the worst perception with respect to their health, with physical well-being and particularly their perception of themselves being the two dimensions with the least favorable evaluation. This perception of themselves constitutes a warning signal for health authorities, given that it could be indicating dissatisfaction of women with their own bodies and, hence, a higher risk of the onset of inadequate nutritional habits [41]. In synthesis, the lower SRH during the school stage could certainly be related to the complexity that characterizes this stage of development, especially in girls from Bogotá, Colombia.

To date, there are few studies that have analyzed the association between SRH and fitness in a young population, including the cardiorespiratory capacity. Häkkinen et al. [42] found a direct association between SRH, physical activity and CRF in a study of 727 youths. This study shows that healthy values of CRF increased the self-perception of well-being and organic health. In keeping with this, Kantoma et al. [5] showed that SRH measured as the question: how do you consider your state of health? And the CRF, estimated using a cycle-ergometer, was associated with better scores in the SRH in Colombia, which is a result that agrees with our study. We found that an unhealthy CRF in children and adolescents is a predictive factor of physical well-being [17,18]. It is important to delve into the components of SRH to detect risk factors associated with enduring future diseases with the aim of implementing different interventions with a perspective of primary health care.

The mechanism between SRH and better CRF is not well established. One plausible explanation relates to the afferent information that conveys messages from the organism to the brain [43]. These messages are usually not brought to consciousness because they function at lower levels of the central nervous system. Another theory explaining a person’s perceptions of their health involves a family of proteins called cytokines [44]. Research is beginning to show that the subclinical inflammatory process and certain cytokines are associated with tiredness, depressive mood, pain, irritability, and poor appetite [45].

This study contained a series of limitations that must be described. The first limitation is inherent to its cross-sectional nature and type of sampling, which does not permit evaluating causality relationships. Second, it would be fitting to broaden the population object of study to different age brackets or private establishments. The reason for having selected a sample between 9 and 17 years of age is due to the variability we can find in physical activity habits during these ages. Third, the study did not evaluate levels of physical activity of the youth, factors that can modulate the results of this work. Fourth, although the evaluation of CRF in children and adolescents was carried out through a valid and standardized test in this population [19], we must bear in mind that the CRF is partially genetically determined [46]. A previous study [47] has tested the degree of agreement between various equations used to estimate VO_2_peak and the actual VO_2_peak. The equation used to estimate VO_2_peak in this study may have underestimated CRF by up to 12% relative to other methods and therefore may have, in isolation, inflated the prevalence of unhealthy aerobic capacity [18,48,49]. Therefore, we considered our low CRF estimates to be conservative. Furthermore, cutoffs proposed were very like the interim international criterion-referenced standards of 35 and 42 ml·kg^−1^·min^−1^ for girls and boys, respectively, to identify children and youth at risk of poor health, raising a clinical red flag [18,50]. However, there are no arguments to believe that the relationships described occur exclusively in the population from our sample, given that we observed convergence of the results with data described in other national and international studies [29,30,31,32,34], hence, the results obtained herein are not compromised.

The strengths of this research broaden the scope regarding SRH in children and adolescents because both age groups were included and the methods used did not refer to specific diseases, like diabetes and myopia, as reflected in other studies [2,10,11]. To our knowledge, this is the first study to demonstrate positive associations of CRF with SRH in Latin-American schoolchildren in a large birth cohort with objective measurements of CRF. Another strength of this study lies in the large, unselected population sample. Furthermore, it approaches specific symptoms and their frequency, which permits better guidance of the specific health needs of children and adolescents from the Latino population. Nevertheless, longitudinal studies are needed to measure other confounding factors that can interfere with the interpretation of the results, like ethnicity, socioeconomic level, physical activity levels, etc. 

## 5. Conclusions 

In summary, SRH was associated with CRF in children and adolescents of both genders. Early identification of children and adolescents with low CRF levels will permit interventions to promote healthy behaviors and prevent future diseases during adulthood. 

## Figures and Tables

**Table 1:** Characteristics of among a population-sample of Schoolchildren in Bogota, Colombia.

Characteristics	Girls	Boys	Overall
	(*n* = 4140)	(*n* = 3280)	(*n* = 7402)
Age (years)	12.8 (2.4)	13.1 (2.4) *	12.8 (2.3)
Body mass (kg)	44.9 (11.4)	45.3 (13.2) **	44.6 (12.3)
Height (m)	1.47 (0.10)	1.56 (0.14) *	1.49 (0.12)
BMI (kg/m^2^)	20.9 (3.5)	19.7 (3.3) *	19.7 (3.4)
VO_2_peak (mL·kg^−1^·min^−1^) ^a^	38.7 (4.8)	42.9 (5.2) *	40.2 (5.3)
Shuttles (total count)	21.3 (11.3)	36.6 (20.0)*	28.9 (17.9)
Stage (last completed)	2.7 (1.3)	4.4 (2.2) *	3.6 (2.1)
Running speed at last completed shuttle (km·h^−^^1^)	9.0 (1.8)	9.9 (2.1) *	3.7 (2.2)
FITNESSGRAM^®^ (VO_2_peak fitness zones) n, [%] ^b^			
Needs improvement	1062 (25.6)	551 (16.7) *	1613 (21.8)
Low CRF (unhealthy)	1407 (33.9)	924 (28.1) *	2331 (31.5)

Note: Frequencies in brackets represent the proportion of the total sample with data for each variable; Significant between-sex differences (*t* student or Chi-square; * *p* < 0.001; ** *p* < 0.01); ^a^ VO_2_peak (mL·kg^−1^·min^−1^) predicted using the Leger et al. equation [27]; ^b^ To classify VO_2_peak, we used the 2011 FITNESSGRAM^®^ standards and healthy fitness zones [28].

**Table 2:** Frequency of self-rated health status in a sample of schoolchildren from Bogotá, Colombia. The FUPRECOL Study.

Question	Frequency	Overall (*n* = 7402)	Girls (*n* = 4140)	Boys (*n* = 3280)	*p* Value
Headache	Rarely or never	4703 (63.3)	2989 (72.2)	1714 (52.3)	<0.001
	Almost every month	600 (8.1)	302 (7.3)	298 (9.1)	0.367
	Almost every week	1392 (18.8)	559 (13.5)	833 (25.4)	<0.001
	More than once a week/almost every day	725 (9.8)	290 (7.0)	435 (13.3)	0.010
Stomach ache	Rarely or never	4837 (65.1)	2935 (70.9)	1902 (58.0)	<0.001
	Almost every month	903 (12.2)	480 (11.6)	423 (12.9)	0.638
	Almost every week	1177 (15.9)	509 (12.3)	668 (20.4)	<0.001
	More than once a week/almost every day	503 (6.8)	215 (5.2)	288 (8.8)	0.125
Backache	Rarely or never	5525 (74.4)	3175 (76.7)	2350 (71.6)	<0.001
	Almost every month	718 (9.7)	406 (9.8)	312 (9.5)	0.026
	Almost every week	785 (10.6)	377 (9.1)	408 (12.4)	0.188
	More than once a week/almost every day	392 (5.3)	182 (4.4)	210 (6.4)	0.444
Feeling-low	Rarely or never	4548 (61.2)	2347 (58.7)	2201 (67.1)	<0.001
	Almost every month	933 (12.6)	497 (12.0)	436 (13.3)	0.644
	Almost every week	1199 (16.2)	799 (19.3)	400 (12.2)	<0.001
	More than once a week/almost every day	740 (9.9)	497 (10.0)	243 (7.4)	0.249
Irritability/bad mood	Rarely or never	3475 (46.7)	2227 (53.8)	1248 (38.0)	<0.001
	Almost every month	955 (12.9)	567 (13.7)	388 (11.8)	0.353
	Almost every week	1776 (24)	865 (20.9)	911 (27.8)	<0.001
	More than once a week/almost every day	1214 (16.4)	480 (11.6)	734 (22.4)	<0.001
Nervousness	Rarely or never	4733 (63.7)	2757 (66.6)	1976 (60.2)	<0.001
	Almost every month	807 (10.9)	489 (11.8)	318 (9.7)	0.359
	Almost every week	1140 (15.4)	555 (13.4)	585 (17.8)	0.059
	More than once a week/almost every day	740 (9.9)	339 (8.2)	401 (12.2)	0.071
Sleeping-difficulties	Rarely or never	5155 (69.4)	2894 (69.9)	2261 (68.9)	0.442
	Almost every month	740 (10)	431 (10.4)	309 (9.4)	0.648
	Almost every week	903 (12.2)	530 (12.8)	373 (11.4)	0.663
	More than once a week/almost every day	622 (8.4)	286 (6.9)	336 (10.2)	<0.001
Dizziness	Rarely or never	5428 (73.2)	3200 (77.3)	2228 (67.9)	<0.001
	Almost every month	696 (9.4)	389 (9.4)	307 (9.4)	0.989
	Almost every week	777 (10.5)	348 (8.4)	429 (13.1)	0.035
	More than once a week/almost every day	511 (6.9)	195 (4.9)	316 (9.6)	0.055

**Table 3:** Relationship between self-rated health status and cardiorespiratory fitness in a sample of schoolchildren from Bogotá, Colombia. The FUPRECOL Study.

Question	Overall (*n* = 7420)			Girls (*n* = 4140)			Boys (*n* = 3280)		
	Healthy (*n* = 4558)	Unhealthy ^a^ (*n* = 2844)	*p* Value	Healthy (*n* = 2381)	Unhealthy ^a^ (*n* =1759)	*p* Value	Healthy (*n* = 2177)	Unhealthy ^a^ (*n* = 1103)	*p* Value
Headache	*n* (%)	*n* (%)		*n* (%)	*n* (%)		*n* (%)	*n* (%)	
Rarely or never	3010 (64.1)	1683 (35.9)	<0.001	1388 (59.8)	933 (40.2)	0.015	1622 (68.4)	750 (31.6)	<0.001
Almost every month	343 (57.3)	256 (42.7)		199 (55.6)	159 (44.4)		144 (59.8)	97 (40.2)	
Almost every week	798 (57.5)	590 (42.5)		510 (53.9)	436 (46.1)		288 (65.2)	154 (34.8)	
More than once a week/almost every day	407 (56.4)	315 (43.6)		284 (57.1)	213 (42.9)		123 (54.7)	102 (45.3)	
Stomach ache									
Rarely or never	3051 (63.3)	1770 (36.7)	<0.001	1474 (59.0)	1023 (41.0)	0.223	1577 (67.9)	747 (32.1)	0.001
Almost every month	538 (59.5)	366 (40.5)		290 (55.4)	233 (44.6)		248 (65.1)	133 (34.9)	
Almost every week	688 (58.6)	486 (41.4)		428 (55.6)	342 (44.4)		260 (64.4)	144 (35.6)	
More than once a week/almost every day	281 (55.9)	222 (44.1)		189 (56.9)	143 (43.1)		92 (53.8)	79 (46.2)	
Backache									
Rarely or never	3468 (63.0)	2041 (37.0)	<0.001	1773 (59.2)	1220 (40.8)	0.006	1695 (67.4)	821 (32.6)	0.049
Almost every month	420 (58.7)	296 (41.3)		217 (55.2)	176 (44.8)		203 (62.8)	120 (37.2)	
Almost every week	447 (56.8)	340 (43.2)		251 (51.2)	239 (48.8)		196 (66.0)	101 (34.0)	
More than once a week/almost every day	223 (57.2)	167 (42.8)		140 (56.9)	106 (43.1)		83 (57.6)	61 (42.4)	
Feeling-low									
Rarely or never	2889 (63.7)	1649 (36.3)	<0.001	1405 (60.0)	935 (40.0)	0.002	1484 (67.5)	714 (32.5)	0.023
Almost every month	581 (62.3)	352 (37.7)		286 (57.7)	210 (42.3)		295 (67.5)	142 (32.5)	
Almost every week	675 (56.4)	522 (43.6)		420 (52.8)	376 (47.2)		255 (63.6)	146 (36.4)	
More than once a week/almost every day	413 (56.3)	321 (43.7)		270 (55.1)	220 (44.9)		143 (58.6)	101 (41.4)	
Irritability/bad mood									
Rarely or never	2183 (63.1)	1274 (36.9)	0.011	1010 (59.7)	681 (40.3)	0.056	1173 (66.4)	593 (33.6)	0.356
Almost every month	600 (63.0)	352 (37.0)		295 (58.9)	206 (41.1)		305 (67.6)	146 (32.4)	
Almost every week	1058 (59.6)	718 (40.4)		596 (54.6)	496 (45.4)		462 (67.5)	222 (32.5)	
More than once a week/almost every day	717 (58.9)	500 (41.1)		480 (57.3)	358 (42.7)		237 (62.5)	142 (37.5)	
Nervousness									
Rarely or never	2944 (62.4)	1774 (37.6)	0.017	1468 (58.0)	1064 (42.0)	0.914	1476 (67.5)	710 (32.5)	0.004
Almost every month	503 (62.3)	304 (37.7)		244 (58.1)	176 (41.9)		259 (66.9)	128 (33.1)	
Almost every week	697 (61.0)	445 (39.0)		407 (57.8)	297 (42.2)		290 (66.2)	148 (33.8)	
More than once a week/almost every day	414 (56.3)	321 (43.7)		262 (56.2)	204 (43.8)		152 (56.5)	117 (43.5)	
Sleeping-difficulties									
Rarely or never	3216 (62.6)	1923 (37.4)	0.030	1682 (59.1)	1163 (40.9)	0.051	1534 (66.9)	760 (33.1)	0.287
Almost every month	452 (61.2)	287 (38.8)		222 (55.6)	177 (44.4)		230 (67.6)	110 (32.4)	
Almost every week	533 (58.9)	372 (41.1)		258 (53.1)	228 (46.9)		275 (65.6)	144 (34.4)	
More than once a week/almost every day	357 (57.7)	262 (42.3)		219 (55.9)	173 (44.1)		138 (60.8)	89 (39.2)	
Dizziness									
Rarely or never	3428 (63.3)	1991 (36.7)	<0.001	1716 (59.5)	1169 (40.5)	0.007	1712 (67.6)	822 (32.4)	0.006
Almost every month	412 (58.9)	287 (41.1)		215 (55.1)	175 (44.9)		197 (63.8)	112 (36.2)	
Almost every week	443 (57.2)	332 (42.8)		262 (52.7)	235 (47.3)		181 (65.1)	97 (34.9)	
More than once a week/almost every day	275 (54.0)	234 (46.0)		188 (53.7)	162 (46.3)		87 (54.7)	72 (45.3)	

^a^ To classify CRF, we used the 2011 FITNESSGRAM^®^ standards and healthy fitness zones (healthy and unhealthy) [28].

**Table 4:** OR and 95% CI for “sometimes symptoms (more than once a week/almost every day)” of self-perceived health according to low cardiorespiratory fitness (unhealthy) in a sample of schoolchildren from Bogotá, Colombia. The FUPRECOL Study.

Question	Boys			Girls		
	OR	95% CI		OR	95% CI	
Headache						
Rarely (almost every month) or never	Reference			Reference		
More than once a week/almost every day	1.30	1.09–1.55		1.19	1.04–1.35	
Stomach ache						
Rarely (almost every month) or never	Reference			Reference		
More than once a week/almost every day	1.31	1.09–1.58		1.10	0.96–1.27	
Backache						
Rarely (almost every month) or never	Reference			Reference		
More than once a week/almost every day	0.95	0.87–1.44		1.26	1.07–1.48	
Feeling-low						
Rarely (almost every month) or never	Reference			Reference		
More than once a week/almost every day	1.29	1.08–1.54		1.28	1.12–1.46	
Irritability/bad mood						
Rarely (almost every month) or never	Reference			Reference		
More than once a week/almost every day	1.04	0.89–1.22		1.17	1.03–1.32	
Nervousness						
Rarely (almost every month) or never	Reference			Reference		
More than once a week/almost every day	1.24	1.04–1.48		1.03	0.90–1.19	
Sleeping-difficulties						
Rarely (almost every month) or never	Reference			Reference		
More than once a week/almost every day	1.14	0.96–1.37		1.20	1.03–1.39	
Dizziness							
Rarely (almost every month) or never	Reference			Reference		
More than once a week/almost every day	1.29	1.05–1.59		1.27	1.09–1.48	

Note: OR: odds ratio; CI: confidence interval. Self-perceived health (“sometimes symptoms” was created, combining the responses of “every week” and “more than once a week/about every day) were included in the logistic regression as a variable category; ORs were adjusted for age and BMI (body mass index).

## Abbreviations

The following abbreviations are used in this manuscript:

FUPRECOL project in Spanish	asociación de la fuerza prensil con manifestaciones de riesgo cardiovascular tempranas en niños y adolescentes colombianos
BMI	body mass index
CRF	cardiorespiratory fitness
HBSC	Health Behavior in School-aged Children
SRH	Self-rated health
VO_2_peak	peak oxygen consumption
TEM	technical error of measurement
